# Identification of latexin by a proteomic analysis in rat normal articular cartilage

**DOI:** 10.1186/1477-5956-8-27

**Published:** 2010-06-05

**Authors:** Elizabeth Pérez, José L Gallegos, Leticia Cortés, Karla G Calderón, José C Luna, Febe E Cázares, María C Velasquillo, Juan B Kouri, Fidel C Hernández

**Affiliations:** 1 Departamento de Infectómica y Patogénesis Molecular, Centro de Investigación y de Estudios Avanzados, Instituto Politécnico Nacional (CINVESTAV-IPN), México; 2 División de Investigación en Salud, Hospital de Ortopedia, "Dr. Victorio de la Fuente Narváez", Instituto Mexicano del Seguro Social (IMSS), México; 3 Unidad de Proteómica Médica, Instituto Nacional de Medicina Genómica (INMEGEN), México; 4 Departamento de Fisiología y Unidad de Microscopia Confocal, CINVESTAV-IPN, México; 5 Unidad de Ingenería de Tejidos, Terapia Celular y Medicina Regenerativa, Instituto Nacional de Rehabilitación (INR), México

## Abstract

**Background:**

Osteoarthritis (OA) is characterized by degeneration of articular cartilage. Animal models of OA induced are a widely used tool in the study of the pathogenesis of disease. Several proteomic techniques for selective extraction of proteins have provided protein profiles of chondrocytes and secretory patterns in normal and osteoarthritic cartilage, including the discovery of new and promising biomarkers. In this proteomic analysis to study several proteins from rat normal articular cartilage, two-dimensional electrophoresis and mass spectrometry (MS) were used. Interestingly, latexin (LXN) was found. Using an immunohistochemical technique, it was possible to determine its localization within the chondrocytes from normal and osteoarthritic articular cartilage.

**Results:**

In this study, 147 proteins were visualized, and 47 proteins were identified by MS. A significant proportion of proteins are involved in metabolic processes and energy (32%), as well as participating in different biological functions including structural organization (19%), signal transduction and molecular signaling (11%), redox homeostasis (9%), transcription and protein synthesis (6%), and transport (6%). The identified proteins were assigned to one or more subcellular compartments.

Among the identified proteins, we found some proteins already recognized in other studies such as OA-associated proteins. Interestingly, we identified LXN, an inhibitor of mammalian carboxypeptidases, which had not been described in articular cartilage. Immunolabeling assays for LXN showed a granular distribution pattern in the cytoplasm of most chondrocytes of the middle, deep and calcified zones of normal articular cartilage as well as in subchondral bone. In osteoarthritic cartilage, LXN was observed in superficial and deep zones.

**Conclusions:**

This study provides the first proteomic analysis of normal articular cartilage of rat. We identified LXN, whose location was demonstrated by immunolabeling in the chondrocytes from the middle, deep and calcified zones of normal articular cartilage, and superficial and deep zones of osteoarthritic cartilage.

## Background

Articular cartilage is a specialized connective tissue and its principal functions are reduction of friction in the joint, resistance to compressive forces and distribution of load [1]. Macromolecules that constitute the extracellular matrix (ECM) of cartilage such as collagens and proteoglycans (PGs) make the reproducibility of protein profiles difficult, interfering with isoelectric focusing (IEF) and masking minor cell proteins [2]. Previous studies reported the use of different selective extraction techniques in human and animal cartilage models, which allowed the rapid and efficient detection of protein expression patterns [3-5].

Proteomic technology is a useful tool in the search for markers aimed at understanding the molecular mechanisms in health and disease conditions. Recently, the protein profile of human articular chondrocytes isolated from cartilage of normal and osteoarthritic individuals was described [6,7]. In other studies where secreted protein patterns in osteoarthritic cartilage explants were compared against normal tissue, increases of collagen type II and activin A were reported [2]. Here we performed the first proteomic analysis of rat normal articular cartilage and, interestingly, latexin (LXN), a protein not previously detected in cartilage, was identified by MALDI TOF/TOF mass spectrometry (MS) from a spot resolved by two-dimensional gel electrophoresis (2-DE). LXN is a 25-kDa protein that functions as a carboxypeptidase inhibitor in mammals and was originally described in the rat lateral neocortex [8]. It has recently been reported that LXN is expressed in proliferating and prehypertrophic chondrocytes during skeletogenesis and bone fracture repair [9]. However, this protein has not been described in mammalian articular cartilage and its role in regulation of chondrocyte function is unknown.

## Results and discussion

### Extraction method and 2-D reference map

Cartilage samples are difficult to fractionate and best results have been observed using a method based on PGs and collagen species removal from protein extracts [2,3]. After test-reported protocols, an improved method resulted in combining purification methods in order to obtain a reliable separation of proteins by 2-DE. First, PGs were eliminated by selective precipitation using cetylpyridinium chloride (CPC). Next, they were precipitated using methanol and, finally, a commercial cleaning-precipitation method was used. With this procedure it was possible to resolve 147 silver-stained spots on each analytical gel loaded with 100 μg of total protein extract. Reproducibility of protein spot profiles was confirmed on triplicate gels from three independent cartilage extracts.

**Figure 1:**
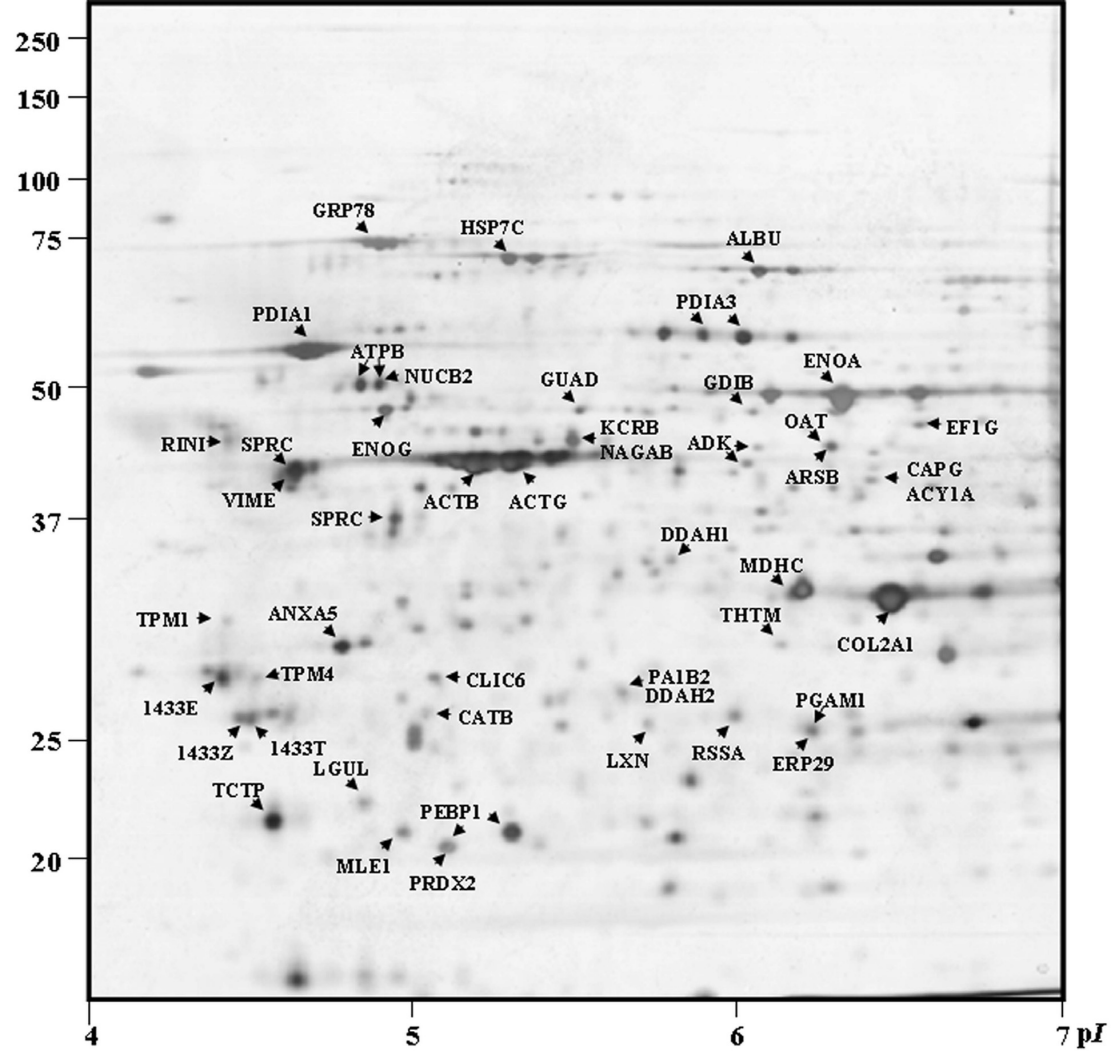
**2-DE map of normal articular cartilage of rat (10% SDS-PAGE, pH 4-7, silver staining)**. Spots are shown according to p*I *and M*w*. Identification of proteins from spots subjected to tryptic digestion followed by MS analysis are shown according to protein names [UniProt Knowledgebase (UniProtKB)/Swiss-Prot] for *Rattus norvegicus *species.

**Figure 2:**
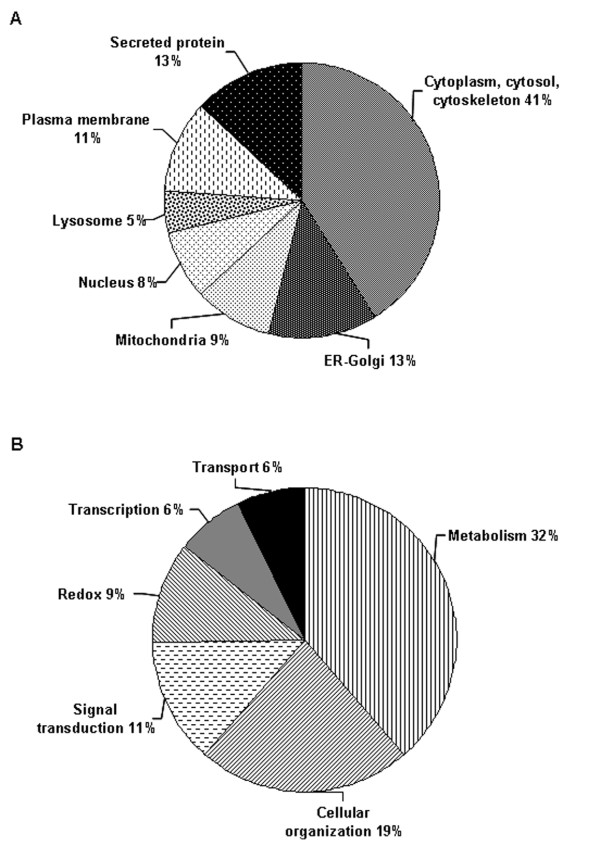
**Gene ontology annotation of the proteins identified in articular cartilage of rat by MS/MS**. Results were obtained from QuickGO for Gene Ontology (GO) database at the EBI at http://www.ebi.ac.uk/QuickGO/. (**A**) Distribution of identified proteins according to subcellular location. (**B**) Functional distribution of identified proteins implicated in biological processes.

A total of 47 proteins from 66 selected and excised spots according to intensity (71.2%) were identified using MS/MS. Proteins identified are listed in Additional file 1, Table S1, indicated on the 2-D reference map (Figure 1), and predicted cell location based on QuickGO for Gene Ontology (GO) database at the EBI is presented http://www.ebi.ac.uk/QuickGO/ (Figure 2A).

The identified proteins were assigned to one or more subcellular compartment such as cytoplasm, soluble cytosol fractions, cytoskeleton, endoplasmic reticulum (ER)/Golgi complex, plasma membrane, mitochondria, lysosome, nucleus and a proportion of secreted proteins.

### Biological function

Proteins categorized according to known biological processes are shown in Figure 2B. For 98% of the proteins, at least one function within the GO database was found. It was observed that a significant proportion of identified proteins are involved in cellular metabolic processes (32%) including enzymes such as esterases, sulfatases, hydrolases, kinases, dehydrogenases, ligases, and lyases. Lactoylglutathione lyase, a catalyst for the conversion of methylglyoxal to D-lactic acid via intermediate SD-lactoylglutation [10], may be involved in the survival of chondrocytes in an anaerobic environment.

A small percentage of identified proteins are involved in other functions including structural organization (19%), signal transduction and molecular signaling (11%), redox homeostasis (9%), transcription and protein synthesis (6%), and transport (6%).

Among the identified proteins, some were already recognized in other studies as OA-associated proteins: enolase, peroxiredoxin, and annexin in human cartilage [11,12].

In addition, a new finding in articular cartilage was the presence of CLIC6 protein, a member of the selective ion channel family [13]. It was previously reported that membrane conductance of rabbit articular chondrocytes was determined by voltage-dependent Cl^- ^channels [14]. Expression of voltage-dependent potassium channels was observed from rat articular chondrocytes, suggesting a role in regulating membrane potential [15]. Furthermore, other studies reported the involvement of ion channels in the processes of proliferation and differentiation of chondrocytes [16,17]. Future studies should demonstrate the role of CLIC6 in the functions of chondrocytes.

Interestingly, in our analysis we found the presence of LXN, a protein not previously reported in articular cartilage. This molecule is a carboxypeptidase inhibitor (CPA1, 2) in rodents, mast cell (CPA3) and CPA4 in humans [18]. LXN has been reported in different tissues such as pancreas, lung, heart, prostate, kidney, ovary and colon [19,20]. In the hematopoietic system, LXN has been reported to influence aging of a negative regulator of stem cell number [21]. Also, it has been suggested that LXN is involved in pain perception according to the location on nociceptive neurons in sensory ganglia [22], in inflammation for its expression on stimulated mouse macrophages [23] and LXN gene expression was identified in acute pancreatitis and inflammatory lung diseases [24]. Moreover, LXN gene was upregulated in BMP-2-treated C2C12 cells, suggesting a role in osteoblast differentiation [25].

### Immunohistochemical detection of LXN

**Figure 3:**
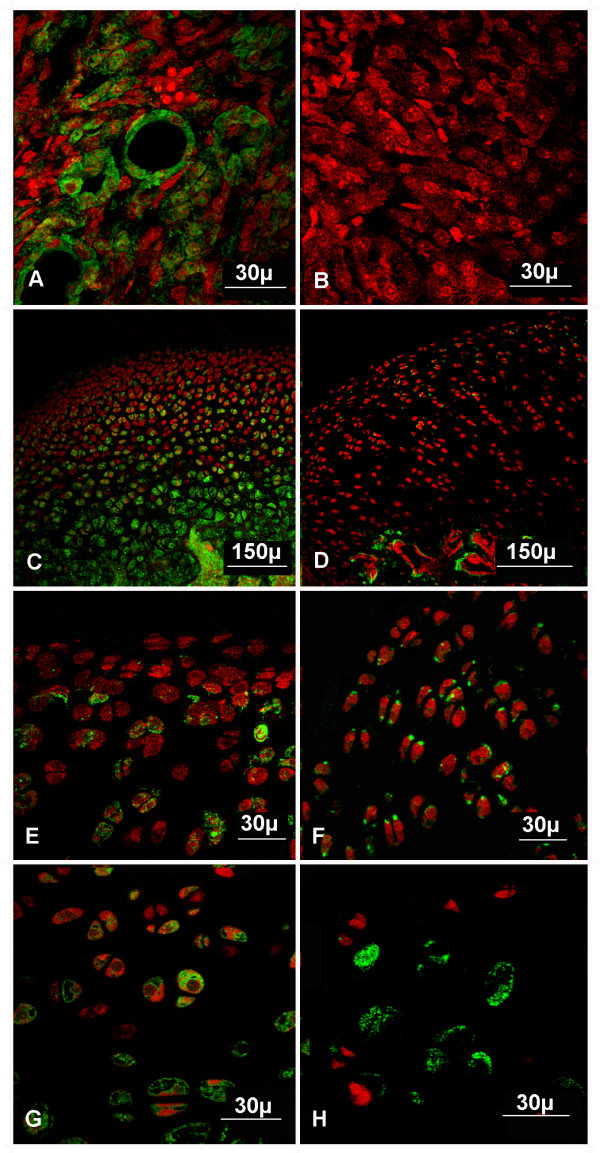
**Immunolabeling of LXN protein in normal articular and osteoarthritic cartilage of rat**. Cryosections were stained with anti-LXN (green) and nuclei were counterstained with propidium iodide (red). (A) Positive control. (B) Negative control. (C, E, G) Normal articular cartilage (total thickness, superficial and middle zones, deep zone, respectively). (D, F, H) Osteoarthritic cartilage (total thickness, superficial and middle zones, deep zone, respectively).

Because it is the first time that LXN, a protein involved in skeletogenesis and growth plaque chondrocyte and osteoblast differentiation [9,26], has been observed in articular cartilage, it was interesting to investigate the localization of this molecule in normal and osteoarthritic articular cartilage of rat. In normal articular cartilage, LXN immunoreactivity was localized predominantly in chondrocytes of middle and deep zones and was also observed in cells of the calcified zone and subchondral bone. Labels were distributed on a granular pattern in the cytoplasm of most chondrocytes (Figure 3). In osteoarthritic cartilage the immunolabeling distribution was confined to chondrocytes of superficial and deep zones in a similar pattern to that of normal cartilage. Further studies will be important to determine if there are statistically significant differences in expression levels of LXN in normal and osteoarthritic cartilage.

Positive and negative controls were performed on rat kidney tissue. The LNX immunoreactivity from positive control was observed in the cytoplasm of the most tubular epithelial cells. Negative control consisted of omission of primary antibody (anti-LXN) and no staining was observed (Figure 3).

Recently, LXN expression in the nucleus and cytoplasm of proliferating and prehypertrophic chondrocytes in the embryonic tibiae and callus during skeletogenesis and skeletal regeneration was described [9]. It was also reported that bone morphogenetic protein-2 (BMP-2) signaling pathway regulates LXN expression during chondrocyte/osteoblast differentiation [9]. To date, LXN expression and localization in mature bone has not yet been determined.

LXN has been reported as a negative regulator of a number of stem cells through decreasing cell replication and increasing apoptosis [21]. Chondrocytes within the cartilage from elderly individuals remain in a quiescent phase of the cell cycle and reduce the potential for growth. This has been associated with changes in telomere length [27]. It would be interesting to investigate whether there is any involvement of LXN in mechanisms of chondrocyte senescence.

On the other hand, it has been suggested that calcification of articular cartilage is not necessarily associated with degenerative conditions in OA, but rather may be a secondary effect of aging [28,29]. Other authors have reported that mineralization of articular cartilage is a fundamental process in OA progression [30,31]. According to our results regarding LXN immunoreactivity in chondrocytes of the deep and calcified zones and subchondral bone, it raises the following question: Could LXN be involved in the process of mineralization of articular cartilage during OA pathogenesis?

## Conclusion

The present study provides the first protein analysis based on 2-DE and MS of normal articular cartilage of adult rat. In addition, this study demonstrated the expression and revealed the immunolocalization of LXN, a protein found in chondrocytes of middle, deep and calcified zones of normal articular cartilage, and superficial and deep zones of osteoarthritic cartilage.

## Materials and methods

### Tissue sampling

Animal samples were obtained following the Guidelines of the Internal Committee for the Care and Use of Laboratory Animals (NOM-069-ZOO-1999). Normal articular and osteoarthritic cartilage was obtained from femoral condyles and tibial plates of male adult Wistar rats (120-150 g) and rats with OA induced by partial menisectomy (20 days after surgery), respectively. This surgical procedure for inducing osteoarthritic cartilage changes has been described elsewhere [32].

Protein preparation was based on selective extraction method with minor modifications [3]. Briefly, samples were rinsed with PBS buffer, frozen in liquid nitrogen, mechanically pulverized and suspended (100 mg cartilage/mL) in extraction buffer (500 mM NaCl, 50 mM HEPES pH 7.2, complete™ protease inhibitor cocktail, Roche, Germany). Samples were stirred overnight at 4°C. Insoluble material was removed by centrifugation (6000 × g, 5 min), and the supernatant was selectively precipitated adding CPC, 1% (w/v). This quaternary ammonium compound forms a water- and alcohol-insoluble complex with glycosaminoglycans [33]. This procedure allowed the removal of anionic macromolecules that interfere with IEF [2,3]. After centrifugation (6000 × g, 5 min) the PG and CPC aggregates were eliminated. Then, supernatant was precipitated with methanol (400 μL methanol/100 μL sample), centrifuged at 14,000 × g for 30 min and suspended again in 4 volumes of methanol. The pellet obtained was resuspended in sample buffer [7 M urea, 2 M thiourea, 4% (w/v) CHAPS, 2% (w/v) immobiline pH gradient (IPG) buffer and 40 mM dithiothreitol (DTT)]. Additionally, we used a method for selective protein precipitation and cleaning (2D Clean-Up Kit, Amersham Biosciences, USA). The precipitate was diluted in rehydration stock solution (7 M urea, 2 M thiourea, 2% (w/v) CHAPS, 0.5% (w/v) IPG buffer and traces of bromophenol blue) supplemented with DTT at 7 mg/2.5 mL of rehydration stock solution. Protein concentrations were measured using 2D Quant Kit (Amersham Biosciences, USA) according to the manufacturer's recommendations.

### 2-DE

Protein extract resuspended in rehydration solution (250 μL) was used to rehydrate Immobiline Drystrip Gels, pH 4-7, 13 cm (GE Healthcare, Sweden) for 18 h at room temperature. Electrofocusing was performed in an Ettan IPGphor 3 Isoelectric Focusing System (GE Healthcare, USA) with an IPG at 16-20 kVh for 5 h. Then, IPG strips were incubated in reducing and alkylating 2-DE equilibration buffer (EB): 6 M urea, 75 mM Tris-HCl (pH 8.8), 29.3% (w/v) glycerol, 2% (w/v) sodium dodecyl sulfate (SDS), traces of bromophenol blue and DTT (100 mg/10 mL EB) or iodoacetamide (250 mg/10 mL EB) for 10 min each, respectively. For SDS-polyacrylamide gel electrophoresis (PAGE), a standard vertical electrophoresis system was used with 10% polyacrylamyde gels (15 cm × 13 cm) using Gibco BRL V16 gel system (Life Technologies, Gaithersburg, MD) according to Laemmli [34]. Gels were stained with Colloidal Coomassie Blue G-250 (Bio-Safe Coomassie Stain, Bio-Rad Laboratories, USA) or silver.

### Image acquisition and data analysis

A digital image of the gels was obtained using scanning densitometry (Image Scanner, Amersham Biosciences Corporation, USA) and analyzed with Image Master 2D Platinum software, version 7.0 (GE Healthcare Life Sciences, Switzerland).

### Spot excision, destaining and drying

For identification of individual 2-D resolved spots, they were excised from gels and transferred to individual Eppendorf tubes. Coomassie Blue dye was removed with 500 μL of 50 mM ammonium bicarbonate (AMBIC) solution in 50% (w/v) acetonitrile (ACN), incubated at 50°C for 5 min and then removed. This procedure was repeated twice. Dehydration was carried out by adding 100 μL of ACN for 5 min at room temperature. The supernatant was then removed and gel fragments were allowed to dry.

### Trypsin digestion and peptide extraction

Peptide digestion was conducted with an in-house-modified protocol [35]. Polyacrylamide pieces containing the spots were rehydrated with sequencing-grade trypsin solution (25 ng/μL, Promega, USA) for 10 min and the excess was removed. Then, 50 mM AMBIC was added to cover the gel completely and incubated overnight at 37°C. Further, 20 μL AMBIC was added to the sample, incubated for 10 min, centrifuged at 14,000 × g for 1 min, and the supernatant was removed and transferred to an empty 0.5 mL plastic vial. Peptide extraction solution [50% (w/v) ACN/0.5% (w/v) trifluoroacetic acid (TFA)] was added, incubated for 10 min at room temperature, vortex mixed and centrifuged at 14,000 × g for 30 sec. The supernatant was carefully collected with a pipette and combined with previous fractions in the 0.5-mL plastic vial. This last step was repeated twice. Protein digests were concentrated/desalted using a solid-phase extraction modified protocol with C18-ZipTips (Millipore Corporation, Bedford, MA, USA). Peptides were eluted in 15 μL of 50% (w/v) ACN/0.1% (w/v) TFA solution.

### MS

Peptides were analyzed using a 4800 *Plus *MALDI TOF/TOF mass spectrometer (Applied Biosystems, Framingham, MA, USA) at the INMEGEN facility. MS/MS spectra were analyzed using Paragon Algorithm (Protein Pilot Software, Applied Biosystems) against the UniProt Knowledgebase (UniProtKB)/Swiss-Prot for *Rattus norvegicus *species plus contaminant protein databases. Search parameters were adjusted for cystein alkylation with iodoacetamide. Confidence interval ≥ 99% was used for protein identification (Unused ProtScore > 2.0) (Additional file 2, Table S2).

### Immunohistochemistry

Full-thickness rat normal and osteoarthritic articular cartilage from weight-bearing areas was fixed with 4% PBS-paraformaldehyde at 4°C, cryosectioned (SM2000 R Sliding Microtome, Leica, Heidelberg, Germany), and mounted on gelatin-coated slides. Sections were incubated overnight at 4°C with an anti-LXN goat polyclonal antibody (1:50, sc-47089, Santa Cruz Biotechnology, Santa Cruz, CA, USA) followed by fluorescein isothiocyanate (FITC)-tagged rabbit anti-goat IgG (1:60; Zymed Laboratories, South San Francisco, CA) for 1 h at room temperature. Nuclei were counterstained with propidium iodide for 5 min (1:1000; Vector Laboratories, Burlingame, CA). Similar preparations where primary antibody was omitted were used for negative controls. Positive controls were conducted on rat kidney tissue.

### Confocal microscopy

Double-immunolabeled sections were viewed through a confocal laser scanning microscope (TCP-SP2, Leica, Heidelberg, Germany) using a 100 × oil-immersion plan Apochromat objective (numerical aperture 1.4). Ten to fifteen consecutive single sections were obtained at 0.8- to 1.0-μm intervals and sequentially scanned for two channels throughout the z-axis of the sample. The resulting stack of images was projected and analyzed on the 2-D plane using a pseudocolor display green (FITC) and red (propidium iodide). Fluorochromes in double-labeled samples were excited at 488 nm (for FITC) and 554 nm (for propidium iodide) wavelengths.

## Competing interests

The authors declare that they have no competing interests.

## Abbreviations

OA: osteoarthritis; MS: mass spectrometry; LXN: latexin; ECM: extracellular matrix; PGs: proteoglycans; IEF: isoelectric focusing; 2-DE: two-dimensional gel electrophoresis; CPC: cetylpyridinium chloride; ER: endoplasmic reticulum; BMP-2: bone morphogenetic protein-2; IPG: immobiline pH gradient; DTT: dithiothreitol; EB: equilibration buffer; SDS: sodium dodecyl sulfate; PAGE: polyacrylamide gel electrophoresis; AMBIC: ammonium bicarbonate; ACN: acetonitrile; TFA: trifluoroacetic acid; FITC: fluorescein isothiocyanate.

## Authors' contributions

EP and LC participated in sample preparation for 2-D and MS, proteomic experiments, image analysis and collection of data. JLG and KGC carried out MS analysis, bioinformatics, and statistical analysis. JCL performed microscopy confocal analysis. FEC carried out the review of the manuscript. MCV facilitated the study samples. JBK and FCH provided direction and funding for this project. All authors read and approved the final manuscript.

## Supplementary Material

Additional file 1 **Functional classification of proteins from normal articular cartilage of rat identified by MS/MS**. Click here for file

Additional file 2 **Detail identified proteins by MS/MS. This information includes protein reported with confidence > 99% (Unused ProtScore > 2.0), with have at least two identified peptides and standard proteins**. Click here for file
