# Supplementary material for: Identification of latexin by a proteomic analysis in rat normal articular cartilage
Source: Proteome Sci. 2010 Jun 5;8:27. doi: 10.1186/1477-5956-8-27 (PMC2904732; doi:10.1186/1477-5956-8-27)
Supplement: Additional file 1 — Functional classification of proteins from normal articular cartilage of rat identified by MS/MS. [file 1477-5956-8-27-S1.DOC]

Table 1. Functional classification of proteins from normal articular cartilage of rat identified by MS/MS.

| Spot name^a)^ | Description protein | Accession #^a)^ | M_r_ (kDa)/ p*I* theor^b)^ | M_r_ (kDa)/ p*I* exp^c)^ | Unused^d)^ | % Seq. Cov.^e)^ |
| --- | --- | --- | --- | --- | --- | --- |
|  | Metabolism and energy (32%) |  |  |  |  |  |
|  | Carbohydrate |  |  |  |  |  |
| LGUL | Lactoylglutathione lyase | Q6P7Q4 | 20.8/5.12 | 22.4/4.9 | 14.96 | 50 |
| NAGAB | Alpha -N-acetylgalactosaminidase precursor | Q66H12 | 46.8/5.55 | 45/5.5 | 6.94 | 10.4 |
|  | Lipid |  |  |  |  |  |
| PA1B2 | Platelet-activating factor acetylhydrolase IB subunit beta | O35264 | 25.5/5.57 | 27.5/5.6 | 15.83 | 43.7 |
|  | Glycosaminglycan |  |  |  |  |  |
| ARSB | Arylsulfatase B | P50430 | 58.9/6.20 | 43.5/6.3 | 2.62 | 1.9 |
|  | Amino acid |  |  |  |  |  |
| ADK | Adenosine kinase | Q64640 | 40.1/5.72 | 43.5/6 | 24.8 | 45.2 |
| ACY1A | Aminoacylase-1A | Q6AYS7 | 45.7/6.03 | 41/6.4 | 8.44 | 13 |
| DDAH1 | NG, NG-dimethylarginine dimethylaminohydrolase 1 | O08557 | 31.3/5.75 | 35/5.8 | 27.97 | 56.8 |
| DDAH2 | NG, NG-dimethylarginine dimethylaminohydrolase 2 | Q6MG60 | 29.6/5.66 | 27.5/5.6 | 11.11 | 31.9 |
| OAT | Ornithine aminotransferase, mitochondrial precursor | P04182 | 48.3/6.53 | 43.5/6.3 | 32.96 | 53.1 |
|  | Glycolysis |  |  |  |  |  |
| ENOA | Alpha-enolase | P04764 | 47.0/6.16 | 49/6.3 | 43.96 | 67.3 |
| ENOG | Gamma-enolase | P07323 | 47.1/5.03 | 47.5/4.9 | 29.17 | 58.1 |
| MDHC | Malate dehydrogenase, cytoplasmic | O88989 | 36.4/6.16 | 33.5/6.2 | 18.7 | 35.9 |
| PGAM1 | Phosphoglycerate mutase 1 | P25113 | 28.8/6.67 | 25.5/6.2 | 7.18 | 14.2 |
|  | Respiratory chain |  |  |  |  |  |
| ATPB | ATP synthase subunit beta, mitochondrial precursor | P10719 | 56.3/5.18 | 50/4.8 | 34.43 | 53.7 |
| KCRB | Creatine kinase B-type | P07335 | 42.6/5.39 | 45/5.5 | 28.01 | 47.2 |
|  | Cellular organization (19%) |  |  |  |  |  |
|  | Cytoskeleton |  |  |  |  |  |
| ACTG | Actin, cytoplasmic 2 | P63259 | 41.7/5.31 | 43/5.3 | 35.17 | 59.7 |
| ACTB | Actin, cytoplasmic 1 | P60711 | 41.7/5.29 | 43/5.2 | 24.45 | 50.7 |
| MLE1 | Myosin light chain 1, skeletal muscle isoform | P02600 | 20.6/4.99 | 21/4.8 | 26.99 | 73 |
| TPM1 | Tropomyosin 1 alpha chain | P04692 | 32.6/4.69 | 31.5/4.4 | 46.12 | 63.4 |
| TPM4 | Tropomyosin alpha-4 chain | P09495 | 28.4/4.66 | 28.5/4.5 | 24.83 | 38.7 |
| VIME | Vimentin | P31000 | 53.7/5.06 | 41.5/4.6 | 3.76 | 4.9 |
|  | Annexin family |  |  |  |  |  |
| ANXA5 | Annexin A5 | P14668 | 35.7/4.92 | 30/4.8 | 35.99 | 62.1 |
|  | ECM |  |  |  |  |  |
| CAPG | Macrophage capping protein | Q6AYC4 | 38.7/6.11 | 41/6.4 | 12.1 | 29.2 |
| CO2A1 | Collagen alpha-1 (II) chain precursor | P05539 | 13.45/8.46 | 32.5/6.5 | 14.95 | 7.6 |
|  | Signal transduction, molecular signaling (11%) | | |  |  |  |
| PEBP1 | Phosphatidylethanolamine-binding protein 1 | P31044 | 20.7/5.47 | 21/5.3 | 22.46 | 76.5 |
| TCTP | Translationally-controlled tumor protein | P63029 | 19.4/4.76 | 21.5/4.5 | 8 | 28.5 |
| 1433E | 14-3-3 protein epsilon | P62260 | 29.1/4.63 | 28.5/4.4 | 27.58 | 63.9 |
| 1433T | 14-3-3 protein theta | P68255 | 27.7/4.69 | 26.5/4.5 | 11.7 | 41.6 |
| 1433Z | 14-3-3 protein zeta/delta | P63102 | 27.7/4.73 | 26.5/4.4 | 26.28 | 55.9 |
|  | Redox homeostasis (6%) |  |  |  |  |  |
| PRDX2 | Peroxiredoxin-2 | P35704 | 21.7/5.34 | 20.4/5.1 | 5.81 | 12.6 |
| PDIA1 | Protein disulfide isomerase precursor | P04785 | 56.9/4.82 | 56/4.7 | 8.7 | 12.4 |
| PDIA3 | Protein disulfide isomerase A3 precursor | P11598 | 56.5/5.88 | 59/5.5-6 | 47.92 | 52.9 |
| THTM | 3-mercaptopyruvate sulfurtransferase | P97532 | 32.9/5.88 | 30/6.1 | 19.23 | 39.4 |
|  | Transcription, protein synthesis (6%) |  |  |  |  |  |
| EF1G | Elongation factor 1-gamma | Q68FR6 | 50.0/6.31 | 47/6.6 | 14.81 | 22.4 |
| RINI | Ribonuclease inhibitor | P29315 | 49.9/4.67 | 45/4.4 | 18.41 | 34.9 |
| RSSA | 40S ribosomal protein SA | P38983 | 32.8/4.80 | 26.5/6 | 20.01 | 50.2 |
|  | Transport (6%) |  |  |  |  |  |
| ALBU | Serum albumin precursor | P02770 | 68.6/6.09 | 68/6.1 | 49.4 | 45.4 |
| CLIC6 | Chloride intracellular channel 6 | Q811Q2 | 64.6/4.29 | 28.5/5.1 | 4.77 | 4.1 |
| GDIB | Rab GDP dissociation inhibitor beta | P50399 | 50.5/5.93 | 47.5/6 | 33.41 | 50.6 |
|  | Others (17%) |  |  |  |  |  |
|  | Stimulus-response, ER stress |  |  |  |  |  |
| GUAD | Guanine deaminase | Q9WTT6 | 50.9/5.56 | 47.5/5.5 | 40.45 | 59 |
| GRP78 | 78kDa glucose-regulated protein precursor | P06761 | 72.3/5.07 | 74.5/4.9 | 56.07 | 44 |
|  | Post- translational processing and destination |  |  |  |  |  |
| ERP29 | Endoplasmic reticulum protein ERp29 precursor | P52555 | 28.5/6.23 | 25.5/6.2 | 24.13 | 54.6 |
| HSP7C | Heat shock cognate 71 kDa protein | P63018 | 70.8/5.37 | 71/5.3 | 30.89 | 28.5 |
|  | Proteolysis |  |  |  |  |  |
| CATB | Cathepsin B precursor | P00787 | 37.4/5.36 | 26.5/5 | 2.98 | 5 |
|  | Ossification |  |  |  |  |  |
| SPRC | SPARC precursor | P16975 | 34.3/4.84 | 41.5/4.6 | 26.13 | 45.4 |
|  | Carboxipeptidase inhibitor |  |  |  |  |  |
| LXN | Latexin | Q64361 | 25.5/5.77 | 26/5.7 | 15.64 | 41.3 |
|  | Unknown biological function |  |  |  |  |  |
| NUCB2 | Nucleobindin-2 precursor | Q9JI85 | 50.0/5.02 | 50/4.9 | 7.03 | 11.4 |

1. Protein name and accession number according Uniprot_sprot database, species *Rattus norvegicus*.
2. Theoretical M_r_ and p*I* according protein sequence.
3. Experimental M_r_ and p*I* calculated by analysis of the gel image with ImageMaster 2D Platinum, Melanie 7.0 software.
4. Protein identification with confidence >99% (Unused ProtScore >2.0). Unused ProtScore: measurement of all the peptide evidence for a protein that is not better explained by a higher ranking protein. Proteins with an Unused ProtScore value higher to 2 have a very high confidence level (>99).
5. Sequence coverage for identified protein (peptide confidence ≥95).
